# Number of risk genotypes is a risk factor for major depressive disorder: a case control study

**DOI:** 10.1186/1744-9081-2-24

**Published:** 2006-07-05

**Authors:** Holly A Garriock, Pedro Delgado, Mitchel A Kling, Linda L Carpenter, Michael Burke, William J Burke, Thomas Schwartz, Lauren B Marangell, Mustafa Husain, Robert P Erickson, Francisco A Moreno

**Affiliations:** 1 Interdisciplinary Program in Genetics, Department of Psychiatry, University of Arizona, Tucson, AZ, USA; 2 Department of Psychiatry, College of Medicine, The University of Arizona Health Sciences Center, 1501 N. Campbell Ave. 7-OPC, Tucson, Arizona 85724, Phone (520) 626-6509, Fax (520) 626-6050, USA; 3 Department of Psychiatry, University of Texas Health Sciences Center at San Antonio, San Antonio, TX, USA; 4 Neuroscience Center, National Institute of Mental Health, Rockville, MD, USA; 5 Department of Psychiatry, Butler Hospital Brown University, Providence, RI, USA; 6 Department of Psychiatry and Behavioral Sciences, Kansas University School of Medicine, Witchita, KS, USA; 7 Department of Psychiatry, University of Nebraska Medical Center, Omaha, NE, USA; 8 Department of Psychiatry, New York State School of Medicine, Purchase, NY, USA; 9 Department of Psychiatry, Baylor College of Medicine, Waco, TX, USA; 10 Department of Psychiatry, University of Texas Southwestern Medical Center, Dallas, TX, USA; 11 Departments of Pediatrics and Molecular & Cellular Biology, University of Arizona, Tucson, AZ, USA

## Abstract

**Background:**

The objective of the study was to determine the genetic basis of Major Depressive Disorder, and the capacity to respond to antidepressant treatment. An association study of 21 candidate polymorphisms relevant to monoamine function and the mechanism of antidepressant response was conducted in 3 phenotypically distinct samples: a group with chronic or recurrent depression unable to respond to antidepressants (non-responders) (n = 58), a group capable of symptomatic improvement with or without treatment (responders) (n = 39), and volunteer controls (n = 85). The responders and non-responders constituted a larger group of depressed subjects.

**Methods:**

A candidate gene approach was employed to asses the genetics basis of Major Depressive Disorder. The genotypic frequencies of selected polymorphisms were compared between the controls and depressed subjects. To asses the genetics basis of the capacity to respond to antidepressant treatment, the responders were compared to the non-responders. Candidate genes were chosen based on functional studies and proximity to whole genome linkage findings in the literature. Risk genotypes were identified by previous functional studies and association studies.

**Results:**

A statistically significant difference in genotype frequency for the *SLC6A4 *intron 2 VNTR was detected between the subjects with a history of depression and controls (p = 0.004). Surprisingly, a statistically significant difference was detected between responders and non-responders for the *DRD4 *exon III VNTR genotype frequencies (p = 0.009). Furthermore, a difference between the controls and depressed subjects as well as between the controls and non-responders was detected for the number and distribution of risk genotypes in each group.

**Conclusion:**

An association between several monoamine-related genes and Major Depressive Disorder is supported. The data suggest that the two depressive phenotypes are genetically different, inferring that the genetic basis for the capacity to respond to standard antidepressant treatment, and the genetic susceptibility to Major Depressive Disorder may be independent. In addition, a proof of concept is provided demonstrating that the number of risk genotypes may be an indication of susceptibility of major depressive disorder and the severity of the disorder.

## Background

Depression affects more than 40 million Americans at some time during their lives and represents one of the most debilitating medical conditions. A substantial body of evidence drawn from a range of methods, proband groups, and diagnostic criteria has established that a familial phenotype is present in patients with major depression [1-7]. Despite their limitations, molecular methods such as candidate gene association studies also support the concept of a genetic influence in vulnerability to depression [8,9]. Although genetic studies promise to improve our understanding of the pathophysiological and genetic aspects of the depressive syndrome, such understanding is limited by the genetic complexity and lack of discrete, etio-pathologically related, and homogeneous "depression" phenotypes.

Treatment resistant depression (TRD) is a characteristic of some depression phenotypes. It can range from not being able to respond to one medication, to not clinically responding to electroconvulsive therapy[10]. TRD, or non-response can be viewed as a spectrum of severity of major depressive disorder, as the amount of suffering is greater for those with TRD, than those without[11]. Whether or not TRD is a subtype of depression with a distinct underlying biology is still unknown and under investigation. It is possible that to some extent TRD is a result of hyperfunctioning enzymes that metabolize typical antidepressant medications [12]. In this case, the TRD subjects would have a genetic background that leads to a pharmacokinetic difference from the depressive group which is capable of antidepressant treatment response. This genetic discrepancy between two diagnostically similar groups (though phenotypically difference in terms of response profiles) may be detectable, and would provide insight to the biology of treatment resistant depression.

To explore genetic differences within depression endophenotypes, we genotyped Caucasian subjects with unipolar major depression from two distinct treatment response profiles: those able to respond to treatment, and those not able to respond. The genes being studied encode for receptors, transporters, synthetic or degradation enzymes, transcription factors, and neurotrophic factors related to monoamine function. We hypothesized that the depressive group as a whole would be genetically distinct from the healthy controls. It was predicted that there would be a higher frequency of "risk" genotypes (excessive or deficient in functioning depending on the case) in the depressive groups when compared to the controls.

We report one two single gene findings and one polygenic discovery. A difference in genotypic frequency between the controls and depressed subjects was detected in the serotonin transporter intron 2 VNTR polymorphism. A surprising difference was detected between the responder and non-responders in the dopamine D4 receptor exon III VNTR. Furthermore, we demonstrate that depressed subjects have more risk genotypes that non-depressed subjects, and that non-responders have the most risk genotypes of all our phenotypic groups analyzed.

## Methods

### Subjects

Ninety-seven subjects, aged 18 to 85 years and diagnosed with unipolar major depression (DSM-IV)[13] participated in the study (see Table 1). Fifty-eight of these subjects had participated in a research trial for treatment resistant depression (TRD); selection criteria for this group included clinical diagnosis of chronic or recurrent depression and failure to achieve remission after at least two adequate antidepressant trials as documented by the Antidepressant Treatment History Form (ATHF) [14-20] These subjects are a subgroup of a vagal nerve stimulation (VNS) trial, and express a highly debilitating course of major depressive disorder, and nearly 90% failed to respond to selective serotonin re-uptake inhibitors (SSRIs). Further details about the subjects has been previously described[21]. Thirty-nine others were selected because they had recovered from a major depressive episode (diagnosed according to DSM-III-R criteria), facilitated by the capability to respond to standard antidepressant medication. The response to the antidepressant medication was indicated by a score of ≤14 on the 25 item – Hamilton Depression Rating Scale[22]. The control group consisted of 85 volunteer students from the University of Arizona, who denied a personal history of mental illness based on the mood disorders section of the Structured Questionnaire Interview for DSM-IV-R (SCID) questionnaire[23]. All participants were of European descent.

**Table 1:** Clinical description and demographics of subjects groups

Subject term	Descriptive clinical information	N	% female	Mean years of age (range)
Depressed	Combination of Non-responders and responders (see below)	97	66.7%	46.7 (24–72)
Non-responders (treatment resistant depression)	Clinical diagnosis of chronic or recurrent depression and failure to achieve remission after at least two adequate antidepressant trials as documented by the Antidepressant Treatment History Form (ATHF).	58	61%	47.2 (24–65)
Responders	Clinical diagnosis of major depressive disorder, and capability to respond to standard antidepressant medication.	39	75%	46.1 (25–72)
Controls	Subjects who denied a personal history of mental illness based on the mood disorders section of the Structured Questionnaire Interview for DSM-IV-R (SCID) questionnaire.	85	64.3%	23.1 (18–68)

### Genotyping

The study was approved by each of the participating institutions. All subjects gave written informed consent for genotyping; samples for DNA analysis were obtained from whole blood or cheek cells. PCR-based genotyping was performed at the Laboratory of Molecular Psychiatry of The University of Arizona for 21 genetic polymorphisms. The details of the analyzed polymorphisms can be found in Table 2. Primer sequences, methodology details and allele description can be found in TABLE 1S provided as supplemental information (See additional file 1). Genotypic frequencies did not deviate from expected Hardy-Weinberg frequencies.

**Table 2:** Polymorphism Descriptions

Gene Name	Risk Genotype	Gene function	Type of Polymorphism	Chromosome Location	location in gene; details
*TFAP2B*	ss/sl	transcription factor	(CAAA)_5–6_	6p12–p21.1	intron 2 near 3' splice site of exon 2; SLC6A4, HTR2A, DbH, DRD1, SLC6A3 have Ap2B binding sites
*BDNF*	AA	Neurotrophic factor	G-->A SNP at nucleotide 196; rs6265;	11p13	proBDNF coding region; (val66met)
*SLC6A4_IN/DEL*	ll	Serotonin transporter	44 bp in/del (5-HTTLPR)	17q11.1–q12	Promoter; actually a VNTR (xs-xl)
*SLC6A4_VNTR*	10/12	Serotonin transporter	VNTR of 17 bp element (9–12 copies)	17q11.1–q12	intron 2
*DRD4_IN/DEL*	sl/ss	Dopamine receptor D4	120 bp in/del	11p15.5	5'-UTR
*DRD4_VNTR*	0 or 1 7R	Dopamine receptor D4	48 bp VNTR	11p15.5	exon 3
*HTR2A*	CC/CT	Serotonin post-synaptic receptor 2A	C102T SNP; rs6313	13q14–q21	exon 1
*SLC6A3*	9/9 and 9/10	Dopamine transporter (SLC6A3)	40 bp VNTR (9 and 10 repeats most frequent, but 3–11 copies possible)	5p15.3	3' non-coding region of exon 15
*rs165599 COMT*	GG	CATECHOL-O-METHYLTRANSFERASE	G-->A SNP (MspI); rs165599	22q11.2	3'-UTR; maybe in mRNA
*DRD3*	GG	Dopamine receptor D3	G-->A SNP (Glycine--> serine) (MscI, isoschizomer of BalI) rs6280	3q13.3	exon 1 (N-terminal extracellular domain)
*DRD1*	GG	Dopamine Recptor D1	G-->C SNP (HaeIII)	5q35.1	-1251
*DRD1*	CC	Dopamine Recptor D1	T-->C SNP (Bsp1286I)	5q35.1	1403
*DRD1*	TT	Dopamine Recptor D1	T-->C SNP (HaeIII)	5q35.1	-800
*HTR6*	CC	Serotonin receptor 6A	T267C SNP (RsaI) Tyr(89) silent mutation; rs1805054	1p36–p35	coding region (1st extracellular loop)
*DRD1*	GG	Dopamine Recptor D1	G-->A SNP (DdeI)	5q35.1	-48
*re4680 COMT*	GG	CATECHOL-O-METHYLTRANSFERASE	G-->A SNP (va158met) (NlaIII) rs4680	22q11.2	4th exon (codon 158 or 108)
*MAOA*	≥3.6R combinations	Monoamine Oxidase type A	30 bp VNTR (2R, 3R, 3.6R, 4R, 5R, 7R)	Xp11.23	1.2 kb upstream of coding region; promoter
*HTR1A*	GG/CG	Serotonin Receptor 1A	C-->G SNP (BstF5I) at position -1019 from ATG start site	5q11.2–q13	PCR fragment is from -1158 to -996 from ATG start site strobel, 2003; promoter
*DBH*	TT (A1/A1)	Dopamine-beta-hydroxylase	C-->T SNp at position 1604; R535C (BstUI); rs6271	9q34	exon 11: 142 bp fragment (originally FnuDII digest). A1: 95, 47 bp; A2: 66, 47, 29 bp.
*TPH2*	GG	Tryptophan Hydroxylase 2 (neuronal TPH)	A-->G; rs1386494	12q21.1	intron 5
*TH*	0,1	Tyrosine Hydroxylase	VNTR	11p15.5	

### Statistical analysis

Initially, four comparisons were made: between subjects with a history of major depression vs. controls, non-responders vs. controls, responders vs. controls, and non-responders vs. responders. The data presented in this study is a result of a chi-square analysis was used to test for differences in the frequency of genotype polymorphisms between the depressive subjects and healthy controls as well as between the responders and non-responders. The remaining two comparisons (responders vs. controls, non-responders vs. controls) is available upon request. All significance tests (aside from QVALUE) were done with SPSS version 14. Genotypic frequency differences were analyzed by Chi-Square tests. False discovery rate analysis was used to control for multiple comparisons between groups and number of polymorphisms. The q-values[24] are reported with all p values ≤0.05. A q-value is an indication of the percent of time a Type I error would be committed if a corresponding p-value was considered as statistically significant. For example, we accepted a false discovery rate of 15%, thus based on the value distribution of the present dataset, a value of q ≤0.137 was considered statistically significant, however, this still implies that 13.7% of the time when we accept a corresponding p-value as statistically significant, an error would be made. The statistical testing for each polymorphism was between a risk genotype determined *a priori *to testing (see Table 2), and the remaining genotypic groups. The mean number of risk genotypes among the phenotypic groups was analyzed using independent samples t-tests. We chose this approach because we had hypotheses that the history positive group would have more risk genotypes that the history negative, and likewise, that the non-responders would have more risk genotypes that the non-responders.

## Results

Table 3 provides the p-value, and corresponding Q-value, for all the polymorphisms tested. A multiple comparison correction (Q-value) was used to decrease the number of false discoveries in the analysis. Genotype frequency differences were statistically significant for the serotonin transporter gene intron-2 VNTR (STin2) between subjects with a history of depression and controls (p = 0.004). The disparate genotypic distribution exists in the heterozygote and 12-repeat homozygote groups. Over 50% of the controls fall into the 12/12 genotypic group, whereas just less than 30% of the depressed individuals do. Likewise, over half of the depressed subjects fall in the heterozygote group, whereas just over 30% of the controls do. The comparison between the depressed and non-depressed subjects also revealed that the *DRD3 *and two *COMT *polymorphisms were close to being statistically significant but did not hold up to multiple comparison correction. A statistically significant difference was detected in the genotypic frequency distributions between the non-responders and the responders of the *DRD4 *exon 3 VNTR (p = 0.009). The genotypic group with zero-7-repeat alleles was largely over-represented in the non-responders, compared to 38% of the responders. Likewise, the homozygous 7-repeat genotypic group had twice the amount of responders compared to non-responders.

**Table 3:** P and Q-values for trending and statistically significant (after multiple comparison correction *) polymorphisms

	Depressed vs. non-depressed		Responders vs. non-responders	
Polymorphism	p-value	q-value	p-value	q-value

rs6280 *DRD3*	0.055	0.264	0.439	0.555
rs4680 *COMT*	0.033	0.239	0.986	0.77
rs165599 *COMT*	0.041	0.239	0.251	0.482
STin2 VNTR *SLC6A4*	0.004*	0.137	0.749	0.699
exon 3 VNTR *DRD4*	0.316	0.482	0.009*	0.137

**Figure 1:**
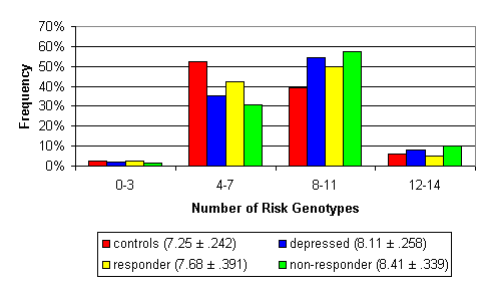
Distribution of risk genotypes for all phenotypic groups (mean # risk genotypes ± SEM).

Figure 1 shows the distribution of the number of risk genotypes for all phenotypic groups. The mean number of risk genotypes (see Figure 1) is statistically significantly different between the controls and depressed subjects (p = 0.017) as well as between controls and non-responders (p = 0.005).

## Discussion

The genetic susceptibility to major depression was tested using genotype frequency comparisons between subjects with a history of depression and controls. We detected three polymorphisms (*SLC6A4 *intron 2 VNTR, 2 *COMT *polymorphisms) which may contribute to the genetic susceptibility to major depression, however, only one (*SLC6A4*) which held up to multiple comparison corrections.

The comparison between the non-responders and responders is a method to detect the genetic basis for antidepressant response, in general. Out of the 21 polymorphisms tested, only one (*DRD4 *exon 3 VNTR) was statistically significant. This is compared to the 3–4 polymorphisms which demonstrate a genetic susceptibility to depression. It was unpredicted that the genetic basis for antidepressant response may differ from the genetic basis of susceptibility to major depression.

We further demonstrated that the number of risk genotypes is not consistent across phenotypes. The non-responders have the most risk genotypes, followed by (in decreasing order) the depressed group as a whole, the responders, and the controls. This supports the notion that Major Depressive Disorder is polygenic, and that the number of risk genotypes may be an indication of the susceptibility to Major Depressive Disorder as well as the severity of the disorder.

In retrospect, we recognize that much of our current understanding of the pathophysiology of depressive disorders has been inferred from the prevailing hypotheses of mechanisms for antidepressant action. Accordingly, candidate genes selected for their function are commonly associated with monoamine function or their putative intracellular responses to neurotransmitter activation. The neurobiology of treatment resistance is not well understood; it may represent an extreme phenotype along a unique pathological continuum of depression. This interpretation may be supported by the fact that the usual monoamine-based treatment interventions, by definition, provide little or no benefit to patients with treatment resistant depression.

The Hamilton Depression Rating Scale has been for decades the gold standard of depression symptoms quantification and has been utilized in >95% of antidepressant treatment studies. There have been lately a number of concerns primarily about the scale's ability to reliably quantify antidepressant responses given the multi-dimensionality of the scale, which may non-specifically determine response in individuals treated with sedating agents such as TCA's. In the other hand, given the number of non-melancholic specific items, it may be less sensitive at detecting an antidepressant response. It is possible that alternative tools such as the Bech Rafelsen or the Montgomery Asberg Depression Rating Scale among others may more accurately reflect antidepressant responses leading to a more reliable detection of therapeutic effects.

This pilot study provides a proof of concept that two depressive phenotypes (antidepressant response and non-response) may be subtypes of Major Depressive Disorder. In terms of medication response, these patients are clearly distinct from each other, thus it is logical to propose their genetic constitution may be distinct. The polymorphisms analyzed in this study is by no means an exhaustive list of polymorphisms which play a role in Major Depressive Disorder, however it is a fairly representative list for the monoamine related polymorphisms thought to confer susceptibility to major depression. It is true that the mean age of the controls is younger compared to the depressed groups, thus they have not necessarily passed the age of onset typical of major depressive disorder. As a result, we may be committing a type I error in the analyses, and be missing or underestimating genetic differences that exist between the controls and depressed subjects, however, we are confident in the effect that we did detect. A large number of comparisons between groups for each polymorphism were initially performed in this study. To correct for false discovery, a q-value was determined, which provided an indication of statistical significance. We accepted a q-value less than 15%, which indicates that 15% of the time we considered out findings to be significant, we would be incorrect. Given that this is a pilot study with relatively small sample size, our single-polymorphism findings may be false positives, and thus necessitate replication in an independent and larger sample. Polymorphisms in alternative neurotransmitter systems, neurotrophic pathways, neurosteroids and antidepressant metabolic pathways (e.g. cytochrome p450 factors) should be the focus of further research.

## Conclusion

Despite the modest sample size, these data support the existence of a genetic basis to the susceptibility to major depression. Furthermore, depending on the phenotypic definition used in testing, different associations may be detected. Given that we utilized phenotypic definitions based on patters of treatment response, these findings further suggest that the genetic basis for the capacity to respond to monoamine-based antidepressants is different from that of susceptibility to major depression. These data provide a proof of concept that major depressive disorder is a polygenic disorder, and that the number of risk genotypes may be an indication of susceptibility to the disorder and the severity.

## Abbreviations

VNTR: variable number of tandem repeats

SNP: single nucleotide polymorphism

TRD: treatment resistant depression

DRD4: dopamine receptor D4

ATHF: Antidepressant Treatment History Form

PCR: polymerase chain reaction

DSM: diagnostic and statistical manual

SCID: Structured Questionnaire Interview for DSM-IV-R

## Competing interests

The author(s) declare that they have no competing interests.

## Authors' contributions

HG carried out molecular genetics analyses, data analyses, and drafted the manuscript. FM led in the design, acquisition of support, and coordination of the study. RPE provided mentorship and guidance in the molecular aspects of the protocol. PD contributed the data for the majority of treatment responders, and MK, LC, MB, WB. TS, LM, MH were principal investigators for the study at their respective sites, contributed recruitment of subjects with TRD, and critically revised the manuscript. All authors read and approved the final manuscript.

## Previous presentations

These data have been presented in abstract form at the Annual Meeting of the American College of Neuropsychopharmacology, December, 2004, the Society of Biological Psychiatry, May, 2005 (poster), and West Coast College of Biological Psychiatry, April, 2006 (talk).

**Table 4:** Genotype frequencies for polymorphisms in Table 3 for all phenotypic groups. Risk genotype indiciated by an *.

**rs6280 *DRD3***	**AA**	**AG**	GG*
controls	46 (.55)	34 (.41)	3 (.04)
depressed	47 (.50)	34 (.36)	14 (.14)
responder	18 (.47)	12 (.32)	8 (.21)
non-responder	29 (.51)	22 (.39)	6 (.10)

**rs4680 *COMT***	**AA**	**AG**	GG*

controls	26 (.31)	43 (.52)	14 (.17)
depressed	30 (.32)	34 (.36)	30 (.32)
responder	12 (.32)	13 (.35)	12 (.32)
non-responder	18 (.31)	21 (.37)	18 (.31)

**rs165599 *COMT***	**AA**	**AG**	GG*

controls	46 (.56)	30 (.37)	6 (.07)
depressed	40 (.43)	34 (.37)	18 (.20)
responder	13 (.35)	14 (.38)	10 (.27)
non-responder	27 (.49)	20 (.36)	8 (.15)

**STin2 VNTR *SLC6A4***	**10/10**	10/12*	**12/12**

controls	15 (.18)	25 (.31)	42 (.51)
depressed	18 (.19)	49 (.52)	27 (.29)
responder	8 (.21)	18 (.47)	12 (.32)
non-responder	10 (.18)	31 (.55)	15 (.27)

**Exon 3 VNTR *DRD4***	0–7R*	1–7R*	**2–7R**

controls	56 (67)	22 (.26)	6 (.07)
depressed	55 (.57)	36 (.38)	5 (.05)
responder	15 (.38)	21 (.54)	3 (.08)
non-responder	40 (.70)	15 (.26)	2 (.04)

## Supplementary Material

Additional File 1 **TABLE 1S. Primers and method of analysis for all polymorphisms**. This is a table in word document format describing all primers, method of analysis and allele descriptions for all polymorphisms discussed in the manuscript. Garriock Supplemental Data lists primer sequences and method of analysis for all polymorphisms described in text in a table in a word document. Click here for file
