# Supplementary material for: Number of risk genotypes is a risk factor for major depressive disorder: a case control study
Source: Behav Brain Funct. 2006 Jul 5;2:24. doi: 10.1186/1744-9081-2-24 (PMC1526442; doi:10.1186/1744-9081-2-24)
Supplement: Additional File 1 — TABLE 1S. Primers and method of analysis for all polymorphisms. This is a table in word document format describing all primers, method of analysis and allele descriptions for all polymorphisms discussed in the manuscript. Garriock Supplemental Data lists primer sequences and method of analysis for all polymorphisms described in text in a table in a word document. [file 1744-9081-2-24-S1.doc]

TABLE 1S. Primers and method of analysis for all polymorphisms

| Polymorphism | Primers (with reference) | Method of Analysis | Allele Sizes |
| --- | --- | --- | --- |
| Serotonin transporter intron 2 VNTR (Bellivier et al, 2002; Lesch et al, 1994) | SERTVNTR-F 5’-fam-GCTGTGGACCTGGGCAATGT-3’  SERTVNTR-R 5’-GACTGAGACTGAAAAGACATAATC-3’ | ABI PRISM® Genetic Analyzer (Applied Biosystems) | 290bp=9R  307=10R  341=12R |
| Serotonin transporter promoter 44bp VNTR (Heils et al, 1995; Moreno et al, 2002) | stpr5 5'-GGCGTTGCCGCTCTGAATGC-3'  stpr3 5'-GAGGGACTGAGCTGGACAACCAC-3' | 3% agarose gel electrophresis | 484bp=short  528bp=long |
| Serotonin receptor 2A C102T SNP rs6313 (Warren et al, 1993) | HTR2A-F 5’-TCTGCTACAAGTTCTGGCTT-3’  HTR2A-R 5’-CTGCAGCTTTTTCTCTAGGG-3’ | *MspI* RFLP and 3% agarose gel eletrophresis | 342bp=T  216 +126 bp=C |
| Serotonin Receptor 6 T267C SNP rs1805054 (Tsai et al, 1999) | 5-HT6F 5’-AACTTCTTCCTGGTGTCGCTCTTC-3’  5-HT6R 5’-ATGAGCAGGTAGCGGTCCAGGC-3’ | *RsaI* RFLP and 3% agarose gel electrophresis | 220=T  128 + 92bp=C |
| AP2-β intron 2 VNTR (Damberg et al, 2000) | AP2F 5’- fam-cctaccaccagagccaggaccc–3’  AP2R 5’- cccccctccagaagcattcct–3’  AP2SEQ 5’-GTTCGGAAGCCGGCTCTCTCC-3’ | ABI PRISM® Genetic Analyzer (Applied Biosystems) | 363=5R  367=6R |
| proBDNF val66met G196A SNP rs6265 (Sklar et al, 2002) | BDNF-1PCR 5’-CATCCGAGGACAAGGTGGCTT-3’  BDNF127-R 5’-ACTGTCACACACGCTCAGC-3’  BDNF-1SEQ 5’-GACTCTGGAGAGCGTGAATG-3’ | ABI PRISM® Genetic Analyzer (Applied Biosystems) | SEQUENCED |
| MAOA promoter 30bp VNTR (Deckert et al, 1999; Sabol et al, 1998) | MAOA-F 5’-fam-CCCAGGCTGCTCCAGAAAC-3’  MAOA-R 5’-GGACCTGGGCAGTTGTGC-3’ | ABI PRISM® Genetic Analyzer (Applied Biosystems) | 179bp=2R  207bp=3R  225bp=3.6R  237bp=4R  266bp=5R |
| rs4680 COMT va158met SNP (Rotondo et al, 2002) | rs4680F 5’-CTCATCACCATCGAGATCAA-3’  rs4680R 5’-CCAGGTCTGACAACGGGTCA-3’ | *Nla III* RFLP and 3% agarose gel electrophoresis | 67bp + 22bp + 18bp = A  86bp + 22bp = G |
| rs165599 COMT  3’-UTR SNP (Karayiorgou et al, 1997) | rs165599F 5’-GACATGCTAACCTCTCTGAAC-3’  rs165599R 5’-GTGCAGGTGAACTCAGCTAG-3’ | *Msp I* RFLP and 2% agarose gel  electrophoresis | 759bp = A  385bp + 344bp = G |
| Dopamine Receptor D3  Exon 1 G🡪A SNP rs6280 (Crocq et al, 1992; Joober et al, 2000) | DRD3F 5’-GCTCTATCTCCAACTCTCACA-3’  DRD3R 5’-AAGTCTACTCACCTCCAGGTA-3’ | *Msc I* RFLP and 3% agarose gel electrophoresis | 304bp + 111bp + 47bp = A  206bp + 111bp + 98bp + 47bp = G |
| Dopamine Receptor D4  Exon 3 48bp VNTR (Lichter et al, 1993; Van Tol et al, 1992) | D4-3(F) 5’-GCGACTACGTGGTCTACTCG-3’  D4-12(R) 5’-GGTCTGCGGTGGAGTCTG-3’ | 1.5% agarose gel electrophoresis | 344bp = 2R  485bp = 4R  629bp = 7R |
| Dopamine Receptor D4  5’-UTR 120bp in/del (Seaman et al, 1999) | D4-120F 5’-TTGTCTGTCTTTTCTCATTGTTTCCATTG-3’  D4-120R 5’-GAAGGAGCAGGCACCGTGAGC-3’ | 1.5% agarose gel electrophoresis | 429bp = short  549bp = long |
| Dopamine transporter exon 15 40bp VNTR (Barr et al, 2001; Vandenbergh et al, 1992) | DAT1F 5’-TGTGGTGTAGGGAACGGCCTGAG-3’  DAT1R 5’-CTTCCTGGAGGTCACGGCTCAAGG-3’ | 2% agarose gel electrophoresis | 440bp = 9R  480bp = 10R |
| Dopamine Receptor D1 position -1251, G🡪C SNP (Misener et al, 2004) | D1-1251F 5’-GAGACTGGCGAGGTAACCAG-3’  D1-1251R 5’-TCAGGAGCCTGTGGCAAT-3’ | *HaeIII* RFLP and 2% agarose gel electrophoresis | 191bp + 58bp = G  166bp + 58bp + 25bp = C |
| Dopamine Receptor D1 position -800, T🡪C SNP (Misener et al, 2004) | D1-800F 5’-CTCTCGAAAGGAAGCCAAGA-3’  D1-800R 5’-CGGCTCCGAAACGTTGAG-3’ | *HaeIII* RFLP and 2% gel electrophoresis | 169bp + 112bp = T  143bp + 112bp + 26bp = C |
| Dopamine Receptor D1 position -48, G🡪A SNP (Misener et al, 2004) | D1-48F 5’-ACTGACCCCTATTCCCTGCT-3’  D1-48R 5’-AGCACAGACCAGCGTGTTC-3’ | *DdeI* RFLP and 2% gel electrophoresis | 146bp + 61bp = G  146bp + 42bp + 19bp = A |
| Dopamine Receptor D1 position +1403, T🡪C SNP (Misener et al, 2004) | D1+1403F 5’-TGGAGAAGCTGTCCCCAG-3’  D1+1403R GTACCTTAGTTTCTTAATAGCGA-3’ | *Bsp1286I* RFLP and 2% gel electrophoresis | 189bp = T  167bp + 22bp = G |
| Serotonin Receptor 1A C🡪G SNP at position -1019 from ATG start site (Strobel et al, 2003) | HTR1AF 5’-GGCTGGACTGTTAGATGATAACG-3’  HTR1AR 5’-GGAAGAAGACCGAGTGTGTCAT-3’ | *BstF5I* RFLP and 2.5% gel electrophoresis | 163bp = C  146bp + 17bp = G |
| Dopamine Beta-hydroxylase exon 11 C1603T SNP rs6271 (Perry et al, 1991) | DBHF 5’-CTGCACCTGCCCTCAGGCGTCCGT-3’  DBHR 5’-CTGGAAGCGGACGGCTGAGGACTT-3’ | *BstUI* RFLP and 3.5% gel electrophoresis | 95bp + 47bp = T (A1)  66bp + 47bp + 29bp = C (A2) |
| Tryptophan Hydroxylase 2 (TPH2) rs1386494 intron 5 A🡪G (Zill et al, 2004) | RS1386494-F 5’-GTGACAGAACTAAGTGACTTGG-3’  RS1386494-R 5’-GATATGCTAGTCCTCTGTTGG-3’ | *Hpa II* RFLP and 2% gel electrophoresis | 501bp = A  355bp+ 146bp = G |
| Tyrosine hydroxylase (TH) VNTR intron 1 (Serretti et al, 1998) | TH-F 5’-CAGCTGCCCTAGTCAGCA-3’  TH-R 5’-GCTTCCGAGTGCAGGTCACA-3’ | ABI PRISM® Genetic Analyzer (Applied Biosystems) | 260bp=TH*1=10 repeats  256bp=TH*2=9 repeats  252bp=TH*3=8 repeats  248bp=TH*4=7 repeats  244bp=TH*5=6 repeats |

## REFERENCES FOR POLYMORPHISMS

Barr CL, Xu C, Kroft J, Feng Y, Wigg K, Zai G, Tannock R, Schachar R, Malone M, Roberts W, Nothen MM, Grunhage F, Vandenbergh DJ, Uhl G, Sunohara G, King N, Kennedy JL (2001). Haplotype study of three polymorphisms at the dopamine transporter locus confirm linkage to attention-deficit/hyperactivity disorder. *Biological Psychiatry* 49(4), 333-339.

Bellivier F, Leroux M, Henry C, Rayah F, Rouillon F, Laplanche J-L, Leboyer M (2002). Serotonin transporter gene polymorphism influences age at onset in patients with bipolar affective disorder. *Neuroscience Letters* 334, 17-20.

Crocq MA, Mant R, Asherson P, Williams J, Hode Y, Mayerova A, Collier D, Lannfelt L, Sokoloff P, Schwartz JC, *et al*. (1992). Association between schizophrenia and homozygosity at the dopamine D3 receptor gene. *Journal of Medical Genetics* 29, 858-860.

Damberg M, Garpenstrand H, Alfredsson J, Ekblom J, Forslund K, Rylander G, Oreland L (2000). A polymorphic region in the human transcription factor AP-2β gene is associated with specific personality traits. *Molecular Psychiatry*  5(2), 220-224.

Deckert J, Catalano M, Syagailo YV, Bosi M, Okladnova O, Di Bella D, Nothen MM, Maffei P, Franke P, Fritze J, Maier W, Propping P, Beckmann H, Bellodi L, Lesch KP (1999). Excess of high activity monoamine oxidase A gene promoter alleles in female patients with panic disorder. *Human Molecular Genetics* 8(4), 621-624.

Heils A, Teufel A, Petri S, Seemann M, Bengel D, Balling U, Reiderer P, Lesch KP (1995). Functional promoter and polyadenylation site mapping of the human serotonin (5-HT) transporter gene. *Journal of Neural Transmission General Section* 102(3), 247-254.

Joober R, Toulouse A, Benkelfat C, Lal S, Bloom D, Labelle A, Lalonde P, Turecki G, Rouleau GA (2000). DRD3 and DAT1 genes in schizophrenia: an association study. *Journal of Psychiatric Research* 34, 285-291.

Karayiorgou M, Altemus M, Galke BL, Goldman D, Murphy DL, Ott J, Gogos JA (1997). Genotype determining low catechol-O-methyltransferase activity as a risk factor for obsessive-compulsive disorder. *Proceedings of the National Academy of Sciences* 94(9), 4572-4575.

Lesch KP, Balling U, Gross J, Strauss K, Wolozin BL, Murphy DL, Riederer P (1994). Organization of the human serotonin transporter gene. *Journal of Neural Transmission. General Section* 95, 157-162.

Lichter JB, Barr CL, Kennedy JL, Van Tol HH, Kidd KK, Livak KJ (1993). A hypervariable segment in the human dopamine receptor D4 (DRD4) gene. *Human Molecular Genetics* 2(6), 767-773.

Misener VL, Luca P, Azeke O, Crosbie J, Waldman I, Tannock R, et al (2004). Linkage of the dopamine receptor D1 gene to attention-deficit/hyperactivity disorder. *Molecular Psychiatry* 9: 500-509.

Moreno FA, Rowe DC, Kaiser B, Chase D, Michaels T, Gelernter J, Delgado PL(2002). Association between a serotonin transporter promoter region polymorphism and mood response during tryptophan depletion. *Molecular Psychiatry* 7(2), 213-216.

Neumeister A, Charney DS, Belfer I, Geraci M, Holmes C, Sharabi Y, Alim T, Bonne O, Luckenbaugh DA, Manji H, Goldman D, Goldstein DS. (2005). Sympathoneural and adrenomedullary functional effects of alpha2c-adrenoreceptor gene polymorphism in healthy humans. *Pharmacogenetics and Genomics* 15:143-149.

Perry SE, Phillips III JA, Robertson D (1991). FnuD II RFLP at the human dopamine-β-hydroxylase (DβH) locus. *Nucleic Acids Research* 19:1162.

Rotondo A, Mazzanti C, Dell’Osso L, Rucci P, Sullivan P, Bouanani S, Gonnelli C, Goldman D, Cassano GB (2002). Catechol O-Methyltransferase, Serotonin transporter, and Tryptophan Hydroxylase Gene Polymorphisms in Bipolar Disorder Patients With and without comorbid panic disorder. *American Journal of Psychiatry* 159, 23-29.

Sabol SZ, Hu S, Hamer D (1998). A functional polymorphism in the monoamine oxidase A gene promoter. *Human Genetics* 103(3), 273-279.

Seaman MI, Fisher JB, Chang F, Kidd KK (1999). Tandem duplication polymorphism upstream of the dopamine D4 receptor gene (DRD4). *American Journal of Medical Genetics* 88(6), 705-709.

Serretti A, Macciardi F, Verga M, Cusin C, Pedrini S, Smeraldi E (1998). Tyrosine hydroxylase gene associatied with depressive symtomatology in mood disorder. *American Journal of Medical Genetics (Neuropsychiatric Genetics)* 81:127-130.

Sklar P, Gabriel SB, McInnis MG, Bennett P, Lim YM, Tsan G, Schaffner S, Kirov G, Jones I, Owen M, Craddock N, DePaulo JR, Lander ES (2002). Family-based associtation study of 76 candidate genes in bipolar disorder: BDNF is a potential risk locus. *Molecular Psychiatry* 7, 579-593.

Siffert W, Rosskopf D, Siffert G, Busch S, Moritz A, Erbel R, Sharma AM, Ritz E, Wichmann HE, Jakobs KH, Horsthemke B (1998). Association of a human G-protein Beta3 subunit variant with hypertension. *Nature Genetics* 18:45-48.

Strobel A, Gutknecht L, Rothe C, Reif A, Mössner R, Zeng Y et al (2003). Allelic variation in 5-HT_1A_ receptor expression is associated with anxiety- and depression-related personality traits. *Journal of Neural Transmission* 110: 1445-1453.

Tsai S-H, Liu H-C, Liu T-Y, Wang Y-C, Hong C-J (1999). Association analysis of the 5-HT_6_ receptor polymorphism C267T in Alzheimer's disease. *Neuroscience Letters* 276(2), 138-139.

Vandenbergh DJ, Persico AM, Hawkins AL, Griffin CA, Li X, Jabs EW, Uhl GR (1992). Human dopamine transporter gene (DAT1) maps to chromosome 5p15.3 and displays a VNTR. *Genomics* 14(4), 1104-1106.

Van Tol HH, Wu CM, Guan HC, Ohara K, Bunzow JR, Civelli O, Kennedy J, Seeman P, Niznik HB, Jovanovic V (1992). Multiple dopamine D4 receptor variants in the human population. *Nature* 358(6382), 149-152.

Wang JC, Hinrichs AL, Stock H, Budde J, Allen R, Bertelsen S, et al (2004). Evidence of common and specific genetic effects: association of the muscarinic acetylchoine receptor M2 (CHRM2) gene with alcohol dependence and major depressive syndrome. *Human Molecular Genetics* 13: 1903-1911.

Warren Jr JT, Peacock ML, Rodrigues LC, Fink JK (1993). An MspI polymorphism in the human serotonin receptor gene (HTR2): detection by DGGE and RFLP analysis. *Human Molecular Genetics* 2, 338.

ZIll P, Baghai TC, Zwanzger P, Schule C, Eser D, Rupprecht R, Moller H-J, Bondy B, Ackenheil M (2004). SNP and haplotype analysis of a novel tryptophan hydroxylase isoform (TPH2) gene provide evidence for association with major depression. *Molecular Psychiatry* 9: 1030-1036.
